# Healthcare beliefs and practices of kin caregivers in South Africa: implications for child survival

**DOI:** 10.1186/s12913-021-06357-9

**Published:** 2021-05-22

**Authors:** Khuthala Mabetha, Nicole C. De Wet-Billings, Clifford O. Odimegwu

**Affiliations:** Department of Demography and Population Studies, Schools of Public Health and Social Sciences, 1st Floor, Robert Sobukwe Building, East Campus, University of the Witwatersrand, Johannesburg, 2001 South Africa

**Keywords:** Kin caregivers, Health-seeking practices, Beliefs, Under-five mortality, South Africa

## Abstract

**Background:**

Appropriate health-seeking practices may have a positive influence on child survival, particularly when practiced by kin caregivers of children who are below the age of 5 years. While literature has shown that children who are raised in kinship care often present with poor health outcomes and often have unmet healthcare needs, the health-seeking behaviours and practices of the children’s kin caregivers that ultimately influence these health outcomes remain largely unknown. In this paper, we explored the healthcare beliefs and practices of kin caregivers in South Africa on child survival.

**Methods:**

Overall, 12 structured interviews were conducted with all the participants. Six [6] interviews were conducted in the Eastern Cape province and 6 were conducted in the KwaZulu-Natal province. The sample of participants was obtained by seeking permission from the child welfare authorities in the KwaZulu-Natal and Eastern Cape Department of Social Development (DSD) to assist in identifying a sample of the kin-caregivers who have provided primary care to children below the age of 5. The structured interviews were transcribed and analysed using thematic content analysis. After thematic content analysis was carried out, transcripts were given case numbers and then imported into NViVo version 11 for analysis and interpretation of the findings.

**Results:**

The healthcare seeking behaviours and poor use of healthcare services of the caregivers were largely influenced by their notions and perceptions of health and illness. The notions and perceptions that the caregivers hold about the health statuses of the children placed under their care and illness were found to be largely culturally determined and largely influenced by preconceptions and certain healthcare beliefs. Increased reliance on traditional herbs, Notion of witchcraft and Faith healing emerged as key factors that influence health-seeking practices and beliefs of kin caregivers, thus influencing under-five mortality.

**Conclusion:**

Kin caregivers should be equipped with the necessary guidance, resources and training that facilitate the successful fulfilment of the caregiving role, given the number of unmet needs and challenges that they face. This will in turn translate into positive child health outcomes.

**Supplementary Information:**

The online version contains supplementary material available at 10.1186/s12913-021-06357-9.

## Background

In most societies, the rearing and socialisation of children is not only a responsibility that is assumed by the children’s biological parents, but it also involves other extended adult kin [1–7]. Kin relationships have always been comprised of mothers, fathers, grandparents, siblings, and other extended kin such as aunts and uncles [1, 8–10]. The practice of kinship care, which is the placement of children with other extended kin (other than the biological parents) is a phenomenon that is gradually receiving much attention and focus from child welfare systems, despite its existence in societies for decades [11–14]. It is the largest alternative care option for children who cannot be raised by their biological parents [15–17], and increasingly growing at an accelerated rate compared to traditional foster care [18–23]. It is distinguished according to whom is a blood relative, is related by means of matrimony, adoption, as well as to any individual who has strong familial ties with certain individuals [24].

South Africa is a country known to have diverse family arrangements and household forms, declining marriage rates, and an increase in female-headed households [25]. In South Africa, family structures and responsibilities have changed over time [26, 27]. The family institution is essentially multidimensional in nature. It affects and is affected by the social structure - the various socio-economic, cultural, and political institutions within society [25]. The extended family institution in South Africa has transformed over time, due to the process of urbanisation and modernisation [27]. The traditional family was conventionally the nuclear family and other extended kin. This has now been replaced with new forms of living arrangements due to family instability, death, desertion, and marital dissolution [26]. These changes have resulted in changing family roles. Traditionally, most children who were orphaned would be placed under the care of relatives who assumed full care and responsibility of the children [28, 29]. However, the last few decades have also seen an increase in family arrangements whereby relatives provide primary care (kin-caregivers) to children who are non-orphaned [30–32]. Statistical findings in South Africa have shown that 65% of children (both orphaned and non-orphaned) who do not live with their biological parents, reside with their grandparents (mostly grandmothers), 17% with aunts, 6% with other relatives and a further 1% with non-relatives [33]. Moreover, a comparative study of 49 countries that examined the structure and composition of families indicated that despite the marked increases in family changes on a global level, over the last 50 years, children are most likely to be raised in nuclear families except in South Africa [34].

With the gradual emergence of this form of family structure, considerable efforts have been made by scholars to explore factors that are associated with the shift from parental care to kinship care. Generally, the unusual shape of families in South Africa is partly historical and cultural as it has been a common practice for children to be raised by grandparents [26]. This has been done to strengthen family attachments, intergenerational learning, as well as draw on the ability of non-working extended family members to provide care and support to children. Literature has shown that widespread labour migration which can be largely attributed to the Apartheid era in South Africa, has also contributed to an increased number children receiving primary care from relatives [26]. In fact, even earlier studies have shown that the increased mobility of people within societies has had a major effect on the family institution [35]. Furthermore, research has also found the devastation of the HIV/AIDS pandemic, parental unemployment, parental drug and alcohol abuse, lack of housing, child abandonment, abuse, and neglect to be other significant reasons [36–38].

Families have been largely deemed to play a pivotal role in exposing children to a good social support system and with providing children with adequate primary healthcare [39]. Severe illness resulting from delayed health care seeking represents a large proportion of the mortality burden in South Africa [40, 41]. Appropriate health care seeking behaviours can reduce the occurrence of severe and life-threatening childhood illnesses [41–43]. Empirical evidence indicating that access to healthcare is a major determinant of health, in particular, child health, has been progressively emerging over the decades [44]. In addition, access to healthcare has been found to reduce child mortality and morbidity significantly [40, 45, 46].

The reduction of under-five mortality rates has been a major focus of the Convention on the Rights of Children Initiative [47–50]. The mandate of the Convention of the Rights of Children Initiative is to advocate for a proper family environment. Proper family environment includes alternative care that is conducive towards the development of a child’s full potential [48, 50]. The issue of under-five mortality remains a problem in South Africa, despite the marked decrease in under-five mortality rates in the past decade [51]. Children without parents are important and vulnerable as they are among the most vulnerable group of children and are often exposed to various risks that may be detrimental to their development and wellbeing [52, 53, 54]. As such, children without parents are designated as children with special health care needs [55].

Overall, the general health of children who are raised in kinship care has become a global concern as such children often present with various mental, physical, emotional and behavioural needs [56–67]. Consequently, kin caregivers thus serve as a major determinant of child health outcomes [68–72]. Although kinship care is often viewed to be altruistic and reciprocal, it is not always beneficial for a child who is placed under such care [37]. Previous literature has shown that children who are raised in kinship care have poorer health outcomes than children who are raised in other care contexts [73–77]. In addition, children who are raised by kin caregivers are also less likely to obtain important child healthcare services and measures taken for disease prevention such as check-ups, patient counselling, and screenings [78]. Moreover, children who are raised by kin-caregivers are documented to lack health coverage, lack regular source of care, and are often reported to be in comparatively poor health [55, 79–83].

The unmet health needs of children in kinship care are embedded in their experiences of traumatic events and compounded by their poor access to appropriate health care services [84]. Children raised in kinship care have been reported to have extensive healthcare needs, due possibly to past experiences of neglect [85–87]. Therefore, these children often require an array of services to improve their emotional difficulties [14, 88, 89]. Kin-caregivers have an increased likelihood of being less receptive to the needs of children under their care. They are also less likely to access additional services and are often less likely to have their care regulated by welfare authorities [90]. Further, two older studies that assessed how kin-caregivers address the needs of children in their care found that kin-caregivers are less likely to refer children to healthcare services and often expose children to less extensive services [13, 91].

In the African context, the provision of healthcare services exists in various forms ranging from westernised medical care, self-diagnosis and treatment as well as increased reliance in traditional medicine, which is the predominant form of preferred healthcare service in most African communities [92]. As a result, health-seeking behaviours are often embedded in traditional and cultural beliefs and practices of kin caregivers [93]. The kin caregivers’ beliefs and practices are strongly influenced by their notions and perceptions of health and illness. Notions and perceptions of health refer to how an individual experiences, personally understands and cognitively views or perceives an illness [94–96]. Literature has further shown that the notions and perceptions that kin caregivers have on health or illness have the potential to translate into health behaviours and practices of caregivers that are inadequate and negligent which may often result in adverse outcomes pertaining to a child’s health, development and survival chances [39, 41].

While literature has shown that children who are raised in kinship care often present with poor health outcomes and often have unmet healthcare needs, the health-seeking behaviours and practices of the children’s kin caregivers that ultimately influence these health outcomes remain largely unknown. Most importantly, empirical has research found that children living with relatives often experience a burden of illness and disease that is disproportionally high, and often results in increased risks of mortality [75, 97].

Literature has shown that appropriate health-seeking practices may have a positive influence on child survival, particularly when practiced by kin caregivers of children who are below the age of 5 years [98]. Given this background, it is thus pivotal to examine the health practices of kin caregivers as they may significantly influence the survival, growth, and development of the children under their care. Seen by the development and implementation of the Post-2015 SDGs, that aim to achieve universal health, it is thus important to focus on vulnerable children without parents as their health is a potentially valuable economic investment. Given this background, the research question in this study was what are the health-seeking behaviours and practices of kin caregivers that have influenced under-five mortality among children raised in kinship care in South Africa?

In this paper, we explored the healthcare beliefs and practices of kin caregivers in South Africa and their influence on child survival.

## Methods

### Design

This was a qualitative study which utilised structured interviews using an audio recorder. Qualitative data was collected from kin-caregivers who have experienced a child death. in order to obtain rich narrative findings on the healthcare beliefs and practices of kin-caregivers who had experienced a child death This assisted in further investigating specific themes that emerged from the respondents’ discussions during the interview.

### Setting

South Africa was chosen as the focal area of study based on several central reasons. Firstly, South Africa is among the most populous countries in the African region with respect to its regional youth population as nearly a third (28.6%) of the population is made up of a young or youthful population [99, 100]. Secondly, although under-five mortality rates indicate a marked decrease in South Africa, South Africa fell short of achieving the 2015 Millennium Development Goal target of reducing under-five mortality rates to 20 deaths per 1000 live births [101]. Thirdly, the practice of kinship care is highly prevalent in South Africa with an estimated 64% of non-orphaned children being in the care of relatives [102].

Additionally, it is a common practice in South Africa for children to live separately from their biological parents in the primary care of relatives due to poverty, labour migration, educational opportunities, and cultural factors [102]. Moreover, South Africa was selected as the focal country of study as the South African foster care system is a highly administrative, rigid and complex system due to the legal orders and vast administrative work that need to be carried out to ensure long-term child monitoring [103]. This system has thus resulted in several social workers being unable to provide adequate monitoring to ensure that children are protected from any form of neglect and mistreatment [103]. This often results in the health outcomes of the children and caregivers (particularly home environment in which the children are raised) remaining largely unknown. The two selected research sites were the Eastern Cape and KwaZulu-Natal provinces of South Africa. These provinces were selected as they have the highest recorded rates (over 34%) of children placed under the care of extended family members [25].

### Data collection and participant selection

Overall, 12 structured interviews were conducted with all the participants who had experienced a child death. Six (6) interviews were conducted in the Eastern Cape province and 6 were conducted in the KwaZulu-Natal province. The reasons for this sample size was because the participants are not a homogenous group and the context in which they live may differ. Secondly, the sample size would ensure that each segment of the population of interest is covered in this study thus ensuring sample representativeness. Thirdly, the sample size was derived taking into consideration issues of data saturation. Qualitative data analyses often require a smaller sample size in relation to quantitative analysis as having a large sample size often results in the addition of new participants resulting in similar perspectives which do not improve the explanations of the themes that emerge in the findings.

The sample of participants was obtained by seeking permission from the child welfare authorities in the KwaZulu-Natal and Eastern Cape Department of Social Development (DSD) to assist in identifying a sample of the kin-caregivers who had provided primary care to children below the age of 5 (see Additional file 1: appendix B and C for approval letters). Although the placement of most children who are raised in kinship is recorded in the Child Welfare system, the administrative process of placing children under alternative care depletes the time and resources of social workers to follow up on the wellbeing of children who are placed under such care [33]. Instead, caregivers are encouraged to apply for foster care grants which oftentimes results in social workers not periodically reviewing the placement [33]. Based on this evidence, the scope of this study was limited to kin caregivers as it was envisioned that stakeholders that regulate kinship care may not have comprehensive knowledge about the health-seeking behaviours, challenges and experiences faced by kin caregivers.

### Data analysis

The structured interviews were transcribed and analysed using thematic content analysis which is a qualitative technique that involves transcribing the information obtained from the interviews into readable text [104]. After thematic content analysis was carried out, transcripts were given case numbers and then imported into NViVo version 11 for analysis and interpretation of the findings. Themes emerged through thorough examination of the coded data.

### Ethical considerations

#### Ethical approval

Formal ethical clearance and approval was sought from the Human Research Ethics Committee (HREC) based at the University of the Witwatersrand (PROTOCOL NUMBER H18/10/38). Potential participants were first provided with a participant information sheet that explained the nature and aim of the study. Participants who were recruited in the study were then requested to sign an informed consent form which stipulated the aims of the study, guaranteed confidentiality, provided information on who will have access to the participants’ information, provided information on the storage of data, recording of data and the dissemination of the findings and indicated that the study is voluntary. Participants were further requested to provide verbal consent so that they could fully acknowledge that they understood the purpose of the study. Participants were granted the right to withdraw from the study at any time if they felt the need to do so. Withdrawal or non-participation from the study did not affect the individuals’ access to any services or place them at any disadvantage.

The identity of participants was protected through providing each participant with a participant number and the recordings have been securely stored in a password-protected computer in a secure locked cabinet. To reduce potential distress to participants that could arise while recalling the sad event of the child death during the interview, the assistance of a bereavement counselling organisation was sought prior to conducting the interviews. This was done to ensure that participants who experienced any distress during the interviews would receive assistance from trained bereavement counsellors in the form of grief support services. The services would also assist in the avoidance of potential adverse psychological and emotional health outcomes.

## Results

The demographic characteristics of the 12 participants, the ages at death of the children who died while under the care of a specific kin caregiver as well as the cause of death, are shown in Table 1. Overall, participants were all female and their mean age was 51 years. Majority of the participants had a Grade 12 (Matric) qualification and were unemployed. Generally, the narratives of the participants showed that the under-five children who were placed under their care had ongoing health issues that required the children to visit a healthcare provider regularly. Six (6) children died from respiratory tract infections (pneumonia, bronchiolitis, tuberculosis, and asthma), two (2) died from epileptic seizures, one (1) from HIV/AIDS, one (1) from a cardiovascular illness, one (1) from severe malnutrition and one (1) from a meningeal infection.

**Table 1:** Demographic Profile of interviewed kin caregivers

Participant code	Relationship of kin caregiver to the child	Age of kin caregiver	Kin caregiver’s level of education	Employment status of kin caregiver	Marital status of kin caregiver	Age at death of deceased child	Cause of death of deceased child
Participant-1	Aunt	36 years old	Matric	Employed (casual work)	Single	3 years old	Asthma
Participant-2	Aunt	40 years old	Matric	Unemployed	Married	4 years old	Pneumonia
Participant-3	Grandmother	55 years old	Matric	Employed (casual work)	Married	3 years old	Cardiovascular illness
Participant-4	Sister	29 years old	Grade 11	Unemployed	Married	3 months	Pneumonia
Participant-5	Grandmother	78 years old	Grade 10	Unemployed	Widowed	2 years old	HIV/AIDS
Participant-6	Grandmother	60 years	Matric	Employed	Married	3 years old	Meningeal infection
Participant-7	Grandmother	51 years old	Grade 6	Unemployed	Single	4 years old	Epileptic seizures
Participant-8	Grandmother	56 years old	Grade 10	Self-employed	Single	3 years old	Tuberculosis
Participant-9	Grandmother	81 years old	Grade 7	Unemployed	Married	4 years old	Epileptic seizures
Participant-10	Aunt	41 years old	National Diploma	Employed	Single	2 years old	Asthma
Participant-11	Sister	20 years old	Matric	Unemployed	Single	3 years old	Severe malnutrition
Participant-12	Grandmother	65 years old	Grade 9	Unemployed	Widowed	11 months old	Bronchiolitis

The narratives of the participants showed that kin caregivers held certain misperceptions about the health statuses of children who were under their care and these misperceptions strongly influenced their beliefs about health which ultimately influenced the health outcomes of children under their care. The kin caregivers indicated that their perceptions of health and illness strongly influence their beliefs. Overall, the healthcare seeking behaviours and poor use of healthcare services of the caregivers were largely influenced by their notions and perceptions of health and illness. The notions and perceptions that the caregivers hold about the health statuses of the children placed under their care and illness were found to be largely culturally determined and largely influenced by preconceptions and certain healthcare beliefs. Three major themes that emerged in the narratives were: (1) Increased reliance on traditional herbs, (2) notion of witchcraft and (3) Faith healing.

### Increased reliance on traditional herbs

Some caregivers reported **traditional herbs and herbalists** as trusted sources of healthcare. This increased reliance in traditional medicine has served as a key factor in caregivers having misconceptions about the children’s actual health status and has thus translated into these caregivers seeking less regular medical care from professional healthcare providers. Secondly, the lack of adherence to regular clinic visits was also largely observed to be attributable to low levels of trust in conventional medicine and perceived minimal benefits. Participant 7 was a primary caregiver to a child who died from epilepsy while under her care. She showed increased reliance in traditional healers and misconceptions about health status in her narrative. This reliance has played a major role in her health-seeking behaviours as she had hardly ever taken the child for any clinic and hospital visits. She argued that her daughter who is also a traditional healer who was later committed into a mental institution, left her child under her care. The child’s health suddenly deteriorated following the mother’s institutionalisation. Participant 7 narrated that the child was in very good health, but his health “appeared” to deteriorate due to the occurrence of a dark entity that affected the child. Given this circumstance, Participant 7 perceived the child’s illness as “not requiring medical attention” as it was more “spiritual” and required her to consult a traditional healer that would save the child’s spirit from this dark entity. She further narrated:

“*My grandchild was suddenly experiencing epileptic seizures. When I took him to a traditional healer, the healer told me that we had to buy 4 goats or a cow. So, we went back to the healer with the 4 goats and he performed that ritual so that he could chase away the evil spirits that were attacking the child. He got a whole lot better but later died. It is the evil spirits nothing else” (Grandmother, 51 years old, KwaZulu Natal).*

Another respondent, Participant 1 also shared similar sentiments as Participant 7 by portraying low levels of trust in conventional medicine and having increased faith in the power and usefulness of traditional medicine in treating illnesses. She argued that conventional medicine presented several side effects which altered the child’s health which she perceived to be in very good health, while traditional medicine contains essential herbs that assist in promoting healing. She argued:

“*I have never believed in Western medicine. When the child was still alive and would be ill, I would not even bother giving her any medication or those western pills. I feel that they are the ones that exacerbate any illness. I would just administer an enema on the child or use the sage wood tree when the child has flu. Those are treatments that we were brought up with*” *(Aunt, 36 years old, Eastern Cape).*

Another participant (Participant 6) whose child died from a meningeal infection also portrayed increased faith in indigenous traditional healers. She reported that the child started presenting with some unusual symptoms and took him to a traditional healer who indicated to her that the source of the illness was in actual fact spiritual and was caused by her ancestors as it seemed that they had turned their back on her and had stopped giving her and those close to her protection due to an issue she had left unresolved. This is evident in the following extract:

*“Through my consultation with the traditional healer, I was informed that my ancestors were not happy with me though I was not sure what the cause of their happiness was. The sickness experienced by the child was thus caused by the invocation of the ancestors to punish me for a violation I might have done. The traditional healer then told me that I needed to appease the ancestors by performing a ritual for them. The child unfortunately died while I was busy preparing myself to undertake the ritual.*

These narrative accounts reveal that the effectiveness of western medicine in treating various health conditions is often undermined, with traditional practices and beliefs being commonly prevalent. Furthermore, the non-utilisation of healthcare services leads into increased dependence on traditional remedies. Thus, it can be deduced from these findings that caregivers perceived traditional remedies and cultural practices as a viable option that was effective in promoting optimal health. This then translated into caregivers not seeking medical attention for the children under their care, which resulted in increased mortality hazards even for health conditions that could have been easily managed.

### Notion of witchcraft

Some participants held the belief that the children under their care were in optimal health. However, the shift from good optimal health to poor health was largely attributed to spellcasting and witchcraft. Participant 9 narrated that her daughter who is a Sangoma (traditional healer) had gone for initiation to practice as a traditional healer and left the child under her care. Upon completion, the mother never returned. Participant 9 further narrated that she had heard rumours that her daughter had been against other initiates and now there has been a longing grudge among them regarding power relations. She added that her daughter’s son soon fell ill following this altercation and would just suffer from seizures where the child would experience temporary confusion, a staring spell, lose consciousness and have uncontrollable jerking movements of the arms and legs. She argued that these experiences were all supernatural and the unaccountable misfortune that had befallen the child can be ascribed to the evil influence and practice of sorcery by one or more of the initiates that were in conflict with the child’s mother, in order to pay revenge to the mother. She argued:

*“I truly believe that there was some element of witchcraft to get back at the mother and merely no medical condition. This child has never been sick under my care, nor required me to take him to the clinic for any condition. Ja, witchcraft is real my child. To think they could just choose to hurt the child and not its mother*” *(Grandmother, 81 years old, KwaZulu-Natal).*

This finding gives rise to the view that some kin caregivers do not understand the aetiology of certain health conditions which translates into symptom misinterpretation and various clinical symptoms being attributed to supernatural forces. This in turn, perpetuates the need to consult with traditional healers, thus resulting into caregivers delaying seeking biomedical attention or not seeking any medical attention at all. Thus, beliefs about a particular cause of illness, have a major impact on treatment-seeking decisions.

### Faith healing

The narratives of some caregivers showed that religion and spirituality play a pivotal role in the notions that the kin caregivers hold as well as the medical decisions that they take. The narratives of the caregivers showed that the caregivers relied on faith healing which is a practice that has been adopted in many churches where prophets or prayer healers lead the congregation through prayer. The caregivers further narrated that healing from any form of illness can only be cured by prayer rather than western medicine or even traditional healers and it brings greater optimal health. They also argued that the power of God is greater than anything that exists on this Earth and is very important in healing and achieving very good health in a spiritual, physical, and mental form. This view is illustrated in some narrative accounts below:

Participant 5

“*Prayer and Faith is everything my child. I have witnessed people firsthand who arrived in wheelchairs getting up and walking while one arrives blind and leaves the church seeing once again. Prayer works my child. After church, I would go down on my knees and pray to God because prayer strengthens a person when in trouble. So, God would take over this situation. Although the child did not survive, she was confirmed to have healed by the prayer warriors at church. I just think that the child’s death was simply God trying to show me that the time that we had been borrowed to spend with my grandchild had come to an end and the child had to return home (Heaven*)” *(Grandmother, 78 years old: Eastern Cape).*

Participant 12:

“*I often pray and say, God see this child through. You are the Alpha and the Omega, the Saviour, the healer and the Redeemer. No one other than you can save this child. I believe in you that this child will be healed. Unfortunately, the child died but God’s will has prevailed. He knows the reason and it is him that takes and gives in this world. At least my child is in a better place now (Grandmother, 65 years old, KwaZulu-Natal).*

This brings forth the conclusion that these kin caregivers believe that disease or illness cannot be cured using western medication and requires spiritual intervention. These findings suggest that no western medicine can amount to the power that God has in healing his people. Healing power only comes from God and that the power of faith and prayer promotes a complete healing process as opposed to biomedical applications.

Overall, drawing from all the narratives, it can be deduced that the caregivers’ beliefs about health problems and their perceived benefits of action to tackling these health problems are the main factors that explain their non-engagement in adequate health-seeking behaviour (visit to the clinic or hospital). These outcomes indicate that there is generally poor awareness and knowledge of the seriousness of the children’s illnesses as well as the implications of these illnesses among these kin caregivers. The caregivers’ poor adherence and resistance to conventional medicine thus serves as a key factor that impacts negatively on child health.

## Discussion

The aim of this study was to explore the healthcare beliefs and practices of kin caregivers and their influence on child survival. The overall inference drawn from the findings of this study is that the healthcare beliefs that some kin caregivers possess as well as their health-seeking practices have adverse effects on child survival. Overall, the findings obtained in the narrative accounts of the participants showed that most of the under-five children who were placed under the care of the respective kin caregivers had/have ongoing health issues that required/require the children to visit a healthcare provider regularly. This finding is affirmed by literature that has shown that children who are raised in kinship foster care have increased exposure to chronic and acute health conditions in relation to children who are raised by their biological parents, which makes access to health care services essential to this population of children [105–108]. In addition, children who are raised by kin-caregivers are documented to lack health coverage, lack regular source of care and are often reported to be in comparatively poor health [79].

Appropriate health-seeking practices and activities of primary caregivers play a crucial role in inhibiting and reducing exposure to various childhood diseases, promoting patient medication adherence as well as significantly reducing child mortality and morbidity [109]. Literature has shown that beliefs about health and illness are largely culturally determined [110]. Cultural belief systems significantly influence the health-seeking behaviour of people [111]. The cultural basis of health and illness has been found to be of importance because an individual’s behaviour pertaining to illness and healthcare utilisation is largely influenced by preconceptions and certain healthcare beliefs [111]. A historical review indicated that in order to understand an individual’s health-seeking behaviour, it is imperative to first gain a comprehensive understanding of the individual’s perception of illness [112]. The narratives of some participants showed that they had an increased reliance in the use of traditional herbs as they perceived traditional herbs and herbalists as trusted sources that promote optimal health. This is due to the misperceptions that they held about the health conditions of children that had been placed under their care. This increased reliance in traditional medicine has served as a key factor in caregivers having misconceptions about the children’s actual health status. These findings are thus strongly substantiated by a study which found that despite the effectiveness of western medicine in treating various health conditions, traditional practices and beliefs are commonly prevalent in some communities [113, 114]. This is evident in the increased use of traditional herbs that are highly regarded to be useful and harmless [115]. In addition, the non-utilisation of healthcare services leads into increased dependence on traditional remedies or non-use of medical treatment [116]. This is due to the fact that it is highly probable that caregivers who utilise traditional herbs and perceive them as strengthening the immune system and translating into optimal health, are more likely to delay seeking medical attention or not seeking any medical attention for the children under their care at all. This according to literature, often results in increased levels of mortality even for health conditions that can be easily managed [117, 118]. For instance, one study showed that child mortality is significantly correlated with use of traditional herbal concoctions [119].

Furthermore, some participants held the belief that the children who died while under their care were in optimal health. However, the shift from good optimal health to poor health was largely attributed to spell-casting and witchcraft. This narrative is supported by previous literature which has shown that health-seeking behaviour is influenced by local notions [120]. It has been documented in South Africa that witchcraft is a significant cause of disease and misfortune [121]. In addition, exposure to a specific illness or disease has been perceived to be largely the result of supernatural phenomena, caused by a curse or witchcraft in certain communities [122]. These findings thus suggest that the beliefs that people hold about witchcraft and its influence on health, have adverse effects on health-seeking practices. This in turn, perpetuates the increased reliance in traditional healers who are believed to provide remedies that counteract these supernatural forces, thus translating into reduced use of conventional healthcare services and ultimately adverse health outcomes.

Lastly, the narratives of some caregivers showed that religion and spirituality play a pivotal role in the notions that the kin caregivers hold as well as the medical decisions that they take. The narratives of the caregivers showed that the caregivers relied on faith healing and that healing from any form of illness can only be cured by prayer and the power of God rather than western medicine or even traditional healers and it brings greater optimal health. These narratives are strongly supported by a study which indicated that religion is a factor that plays a pivotal role in the medical decisions of many individuals [123, 124]. Literature has further shown that the first port of call for preventing illnesses and fostering healing is through faith healers [122]. There is also a growing utilisation of faith healing services for curative purposes and health promotion, particularly in various sub-Saharan African regions [121–124]. These outcomes thus propose the argument that individuals view religion as an energising and healing force which restores greater strength through divine healing.

This study has a number of strengths. Firstly, it is the first study in South Africa to explore the healthcare beliefs and practices of relative kin caregivers who are providing care to under-five children and how these beliefs and practices have influenced under-five mortality. It is also the first study, to our knowledge to include kinship carers who are providing care to non-orphaned children who were recruited using a database obtained from the Department of Social Development of all the households who had kinship carers as the resident heads. Lastly, although the participants were obtained in only two of the South African provinces (Eastern Cape and KwaZulu-Natal), the narratives of these participants were supported by existing literature conducted in other contexts which increases the likelihood that the solutions that are implemented in relation to these outcomes may also be applicable beyond the South African context.

Given the sensitive nature of the study, there were some limitations in the qualitative analysis. Three of the participants were hesitant in providing detailed responses during the in-depth interviews and thus probes had to be used. This created difficulties as some of the responses that required further explanations were brief. This could have posed some problems as they may have withheld very important information that could have provided insight into the study and provided informative recommendations to Child Welfare Authorities, healthcare professionals and government at large. Secondly, the narratives of the participants could not be statistically represented or measured.

## Conclusion

The overall inference drawn from this study is that the healthcare practices of kin caregivers are rooted in traditional and cultural beliefs which ultimately influence under-five mortality. This is because they play a crucial role in compromising the ability of kin caregivers to provide adequate care. These circumstances thus expose children placed under such care to various risks that may be detrimental to their health and development. The developmental outcomes that children experience throughout their life trajectories as well as their overall wellbeing are embedded in the provision of adequate care by a receptive caregiver who promotes the healthy upbringing of a child. Such barriers should thus be mitigated by calling upon child welfare authorities to work collaboratively with healthcare professionals in providing joint support to kin caregivers who have assumed the care of children and making access to healthcare services a priority and compulsory for children placed under such care. In sum, kin caregivers have the potential and capability to provide adequate care, as well as a conducive and nurturing environment to the children who are under their care. However, it is imperative that kin caregivers are equipped with the necessary guidance, resources and training that facilitate the successful fulfilment of the caregiving role, given the number of unmet needs and challenges that they face. Most importantly, beliefs of health and illness may play a pivotal role in terms of promotion of health and health education.

## Supplementary Information

**Additional file 1: Appendix A-C.** Structured Interview guide and approval letters.

## Data Availability

Data sharing is not applicable to this article as no datasets were generated or analysed during the current study.
