# Supplementary material for: Healthcare beliefs and practices of kin caregivers in South Africa: implications for child survival
Source: BMC Health Serv Res. 2021 May 22;21:486. doi: 10.1186/s12913-021-06357-9 (PMC8140432; doi:10.1186/s12913-021-06357-9)
Supplement: Supplementary file 1 — Additional file 1: Appendix A-C. Structured Interview guide and approval letters. [file 12913_2021_6357_MOESM1_ESM.docx]

## **Healthcare beliefs and practices of kin caregivers in South Africa: Implications for Child survival**

## **APPENDIX A: Structured Interview guide**


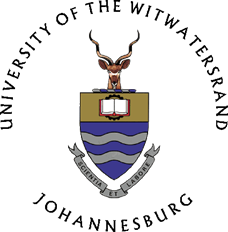


**Interviewee Information**

**Part 1: Demographic information**

Relationship to deceased child: ………………….

Age: ……………………………………………….

Sex: ……………………………………………......

Level of education……………………………….... Occupation………………………………….

Marital status………………………………………

Date of Interview……………………………… Duration of Interview……………………………

Place of Interview………………………………...

Age of deceased at death………………………….

**Part 2: Health-seeking behaviours of kin caregivers and challenges facing kin caregivers**

1. How would you describe your overall wellbeing, physically and emotionally, prior to the child’s death?
2. Would you say your wellbeing changed at all when you started taking care of the deceased child? How so?
3. Has your health ever prevented you from providing care to the deceased child? If so, in what way?
4. Where do you go or whom do you consult when you are ill?
5. How regularly do you visit the clinic and how do you feel about going to the clinic when you are ill?
6. Is it easy to access the clinic? If not, what would you say are the main factors that hinder easy access to the clinic?
7. Did the deceased child have any known ongoing health issues that required the child to visit a healthcare provider regularly? If so, did the deceased child have any health insurance?
8. Please describe any challenges that could have prevented you from seeking regular medical care and treatment for the deceased child.
9. What did you do when the deceased child fell ill?
10. Please explain any other special needs that the deceased had that you believe required specialised care and support?
11. Would you say you felt somehow equipped to care effectively for the deceased child? If so, how? And if not, why?
12. What did you find difficult about providing care to the deceased child?
13. What other challenges (other than health-related challenges) did you experience while providing care to the deceased child?
14. How did you deal with the challenges of providing care to the deceased child?
15. What do you think could have been done to overcome these challenges?
16. What was the most meaningful part of taking care of the deceased child?
17. Moving forward, what resources would you need to take care of any other children that may be in your care, as well as to overcome any challenges you may encounter in your caregiving?

##
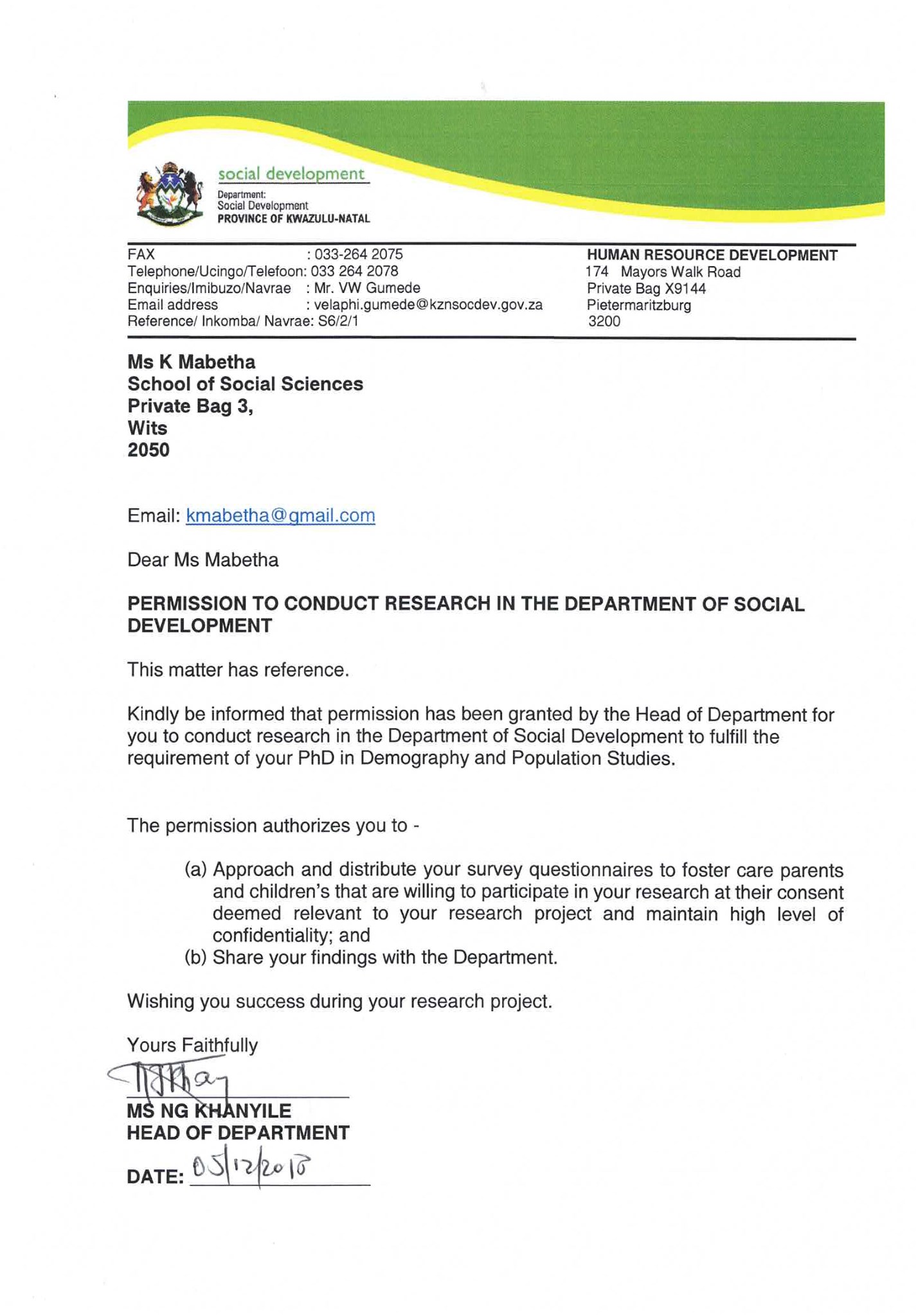
**APPENDIX B: KwaZulu-Natal Department of Social Development Approval Letter**

## **APPENDIX C: Eastern Cape Department of Social Development Approval Letter**

**
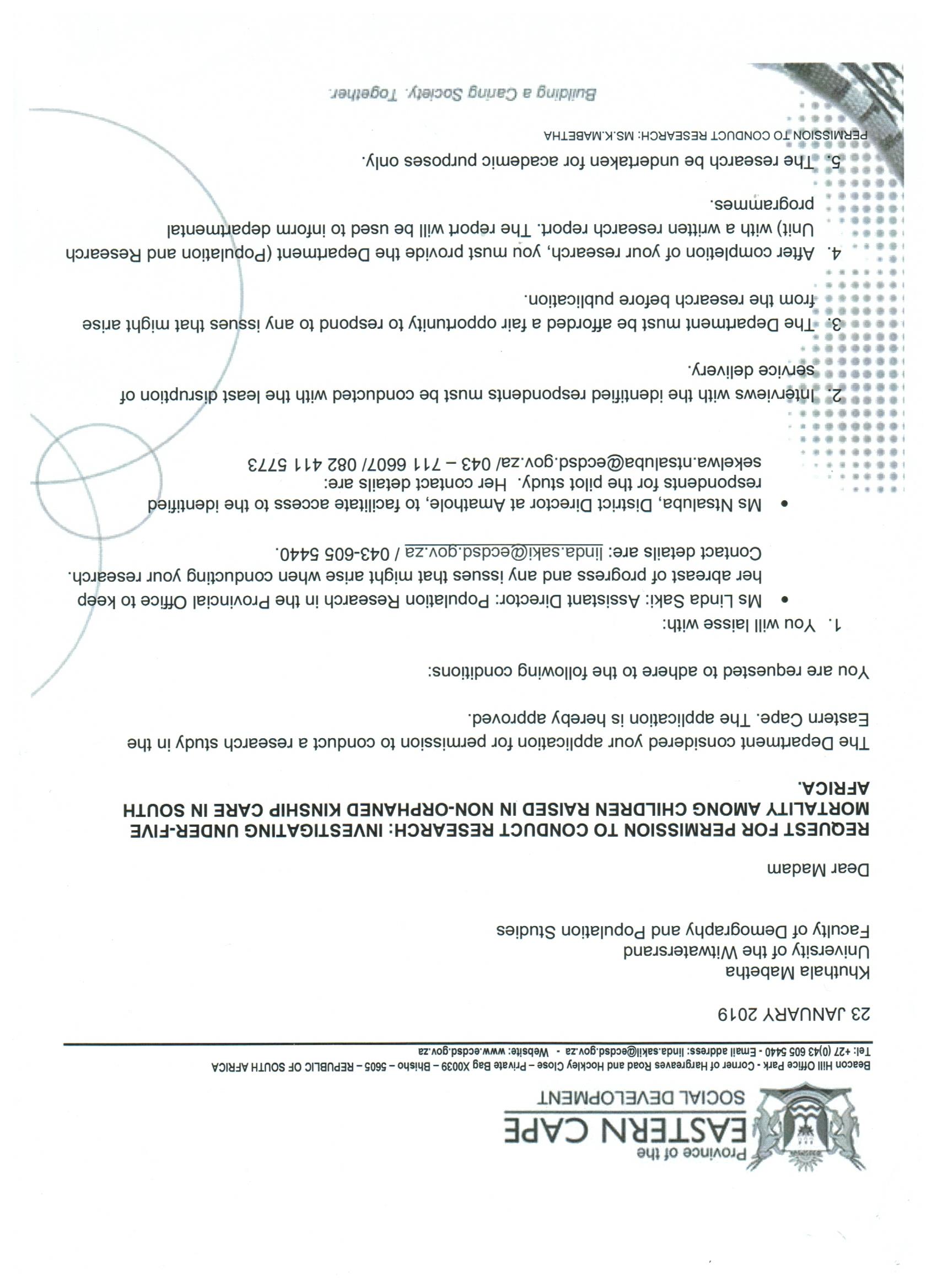
**

**
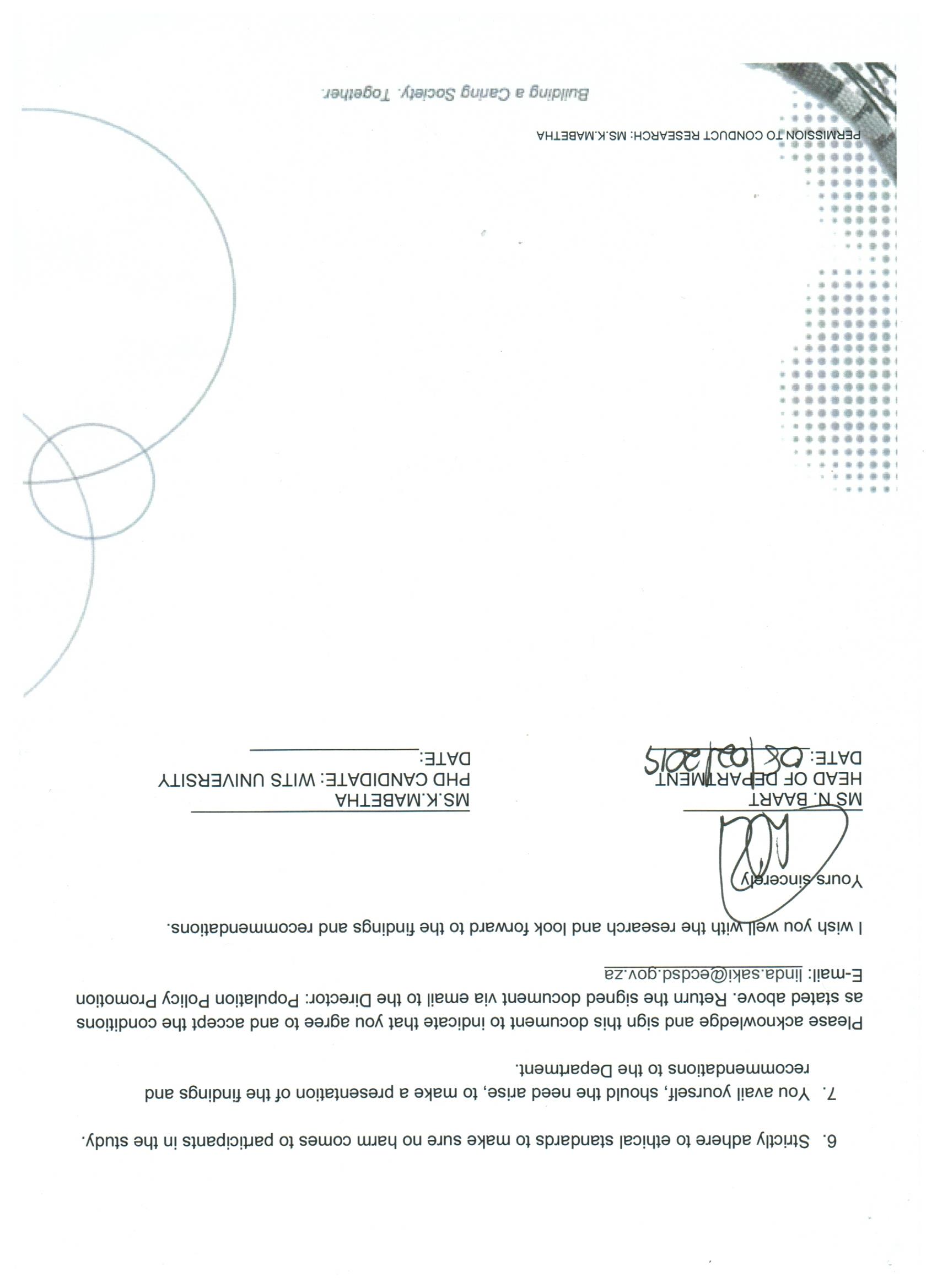
**
